# Prevalence of dental caries and oral hygiene among specially-abled children

**DOI:** 10.6026/9732063002001238

**Published:** 2024-10-31

**Authors:** Aya Misrabi, Dinesh Sharma, Monika Sharma, Shaun Uppal, Mrinalini Sadal

**Affiliations:** 1 General Dentist, Damascus, Syria; 2 Department of Dentistry, Ananta Institute of Medical Sciences and Research Institute, Udaipur, India; 3 MpH-D Ontario, Canada; 4 Private Practitioner, Visakhapatnam, Andhra Pradesh, India

**Keywords:** Dental caries, physical disability, oral hygiene, special children

## Abstract

Dental caries (tooth decay) is a common oral health problem among children, significantly impacting their overall well-being and quality of life. Therefore, it is of interest to find the prevalence of dental caries and oral hygiene status in specially-abled children. This cross-sectional study was conducted to assess the prevalence of dental caries and oral hygiene status in 225 specially-abled children. The participants, aged <18 years, were included in the study. The study included children with physical, intellectual and developmental disabilities, ensuring a diverse representation of conditions that may impact oral health. A total of 225 especially abled children participated in the study, with a mean age of 12.5± 3.4 years. The participants included 130 males (57.8%) and 95 females (42.2%). Children with intellectual disabilities exhibited the highest mean DMFT (Decayed, Missing, Filled Teeth) score (4.2 ± 2.3), with 75% of them affected by dental caries. In comparison, children with physical disabilities had a mean DMFT score of 3.6 ± 1.9 and a caries prevalence of 62%. Thus, the prevalence of dental caries and poor oral hygiene status is notably high among specially-abled children, particularly those with intellectual disabilities.

## Background:

Dental caries is the most widespread chronic disease with a negative impact on global public health by destroying healthy teeth. Worldwide, different findings have shown that most children and an estimated 90% of adults have experienced caries, with the disease most prevalent in the Middle East, Latin America, and South Asia. Cross-sectional analyses compared with a normal population of children have revealed that dental caries in children with disabilities is highly prevalent [1]. This is because most children with disabilities are rarely provided with the required self-care regarding the health of their teeth as most of them lack parental care or the words to express their need for a dentist because of their mental or physical impairments [2]. Germ cavities or caries is one of the most common chronic conditions which affect people all around the globe. They are most common in populations with reduced opportunities for professional oral care and preventive check-ups; children with some form of disability are especially at risk. Such children, categorized under various impaired or disabled, are prone to different forms of health complications and as such may need extra attention because of their disabilities [3^3^]. The need for effective oral hygiene may be challenging for this group due to physical limitations and in some cases, cognitive impairment; access to professional dental care is likely to be restricted; their diet may be unsuitable; and their medication may be detrimental to their teeth [4]. According to the WHO oral health is not only an important part of the general health and well-being of an individual. Nevertheless, children with special needs often face certain challenges in the area of oral health care [5]. Some of these barriers include the inability of the elderly to brush and floss, the lack of a caretaker in the setting performing these functions and physical and or cognitive impairment that hinders an elderly person's ability to attend dental care appointments [6]. Thus, the incidence of dental caries among specially-abled children is higher than that of usual children, and the majority of them are likely to have more severe untreated caries [7]. Other related variables also reveal that oral hygiene status, which is an important determinant of oral health, is also comparatively poor among children with disabilities. Research work has also revealed that especially children with disabilities are prone to having higher retention of plaque, inflammation of the gums, and even periodontal diseases [7, 8]. These conditions are compounded by the problems related to oral hygiene such as the difficulty that caregivers encounter in ensuring the required standard of oral care and, in themselves, the children's difficulties in regulating the routines of oral care. Furthermore, poor dental health characterized by dental caries and other manifestations of poor oral hygiene have implications that extend to the general body as random systemic infections, pain and discomfort which reduce their quality of life [9]. In regards to diet, most CWS (Child Welfare Service) have some sort of dietary restrictions and most are on a very soft and high carbohydrate diet which puts a child at high risk for caries. Furthermore, certain medications that are taken in an attempt to manage their disability, for example, anti-epileptics, precede xerostomia (dry mouth) which inhibits the natural removal process of bacteria and results in tooth decay [10]. These children may not express their pain or discomfort well and this means that dental diseases are not immediately discovered; hence they are not treated. Special needs children require special attention when it comes to oral health, and it is the responsibility of the primary caregivers of the children especially abled kids [11]. However, some barriers may limit the caregivers' capacity to develop appropriate oral health care, for example, the absence of information on oral hygiene practice, or the caregiver may not focus on oral health as compared to other health problems affecting the child [12]. It can lead to a lack of health care dental services which in turn exposes patients to dental caries and poor oral hygiene status. It is therefore important that the caregiver of such a child be educated and trained on the requirements of a child's oral hygiene so that they will be able to effectively monitor their child's dental health needs. Existing literature on the oral health of children with special needs is scarce but increasing and thus it has stressed the need for more extensive studies to detail the extent of dental caries and its risk indicators in these children [2, 3]. Current research has therefore stressed the importance of developing disease prevention intervention that takes into consideration the experiences of the children and their families [13]. Therefore, it is of interest to report the prevalence of dental caries and assess the oral hygiene status among specially-abled children, to identify key factors contributing to their oral health challenges and inform targeted preventive strategies.

## Methodology:

This cross-sectional study was conducted at from Data were collected to assess the prevalence of dental caries and oral hygiene status in 225 especially abled children. The participants, aged <18 years, were included in the study. The study included children with physical, intellectual and developmental disabilities, ensuring a diverse representation of conditions that may impact oral health.

## Inclusion criteria:

[1] Children aged 5-18 years.

[2] Diagnosed with physical, intellectual or developmental disabilities.

[3] No prior major dental treatments (such as full mouth rehabilitation) in the past year.

## Exclusion criteria:

Children with serious medical conditions requiring hospitalization or those unable to participate in the dental examination due to health complications were excluded from the study.

## Data collection:

Data were collected with the approval of the ethical committee of the hospital. The data were collected through a clinical dental examination. Each child underwent a detailed oral examination conducted by a trained dentist. Dental caries was assessed using the WHO's DMFT (Decayed, Missing, Filled Teeth) index. The examination was conducted in natural light using sterilized dental instruments (mouth mirrors and probes). The presence of dental plaque and gingival health was evaluated using the Plaque Index and Gingival Index, respectively. A structured questionnaire was administered to the participants to collect data about the child's oral hygiene practices, dietary habits, frequency of dental visits, and any difficulties encountered in maintaining oral hygiene. The questionnaire also collected demographic details such as the child's age, gender, type of disability, and medication use.

## Data analysis:

Data were analyzed using SPSS v26. Descriptive statistics were used to summarize the prevalence of dental caries and oral hygiene status. The DMFT score was calculated for each child, and the mean DMFT score for the entire sample was reported. Plaque Index and Gingival Index scores were calculated to assess oral hygiene levels. A p-value of <0.05 was considered statistically significant.

## Results:

A total of 225 especially abled children participated in the study, with a mean age of 12.5± 3.4 years. The participants included 130 males (57.8%) and 95 females (42.2%). The children had varying types of disabilities, including physical disabilities (33.8%), intellectual disabilities (45.3%), and developmental disorders such as autism spectrum disorder (20.9%). (Table 1, Figure 1 see PDF and Figure 2see PDF) Children with intellectual disabilities exhibited the highest mean DMFT score (4.2 ± 2.3), with 75% of them affected by dental caries. In comparison, children with physical disabilities had a mean DMFT score of 3.6 ± 1.9 and a caries prevalence of 62%. Meanwhile, children with developmental disorders, such as autism, showed a lower mean DMFT score of 3.1 ± 1.8, with 58% experiencing dental caries (Table 2).

Children with intellectual disabilities had the highest Plaque Index (3.0 ± 0.8) and Gingival Index (2.5 ± 0.7), indicating poor oral hygiene and a greater presence of gingival inflammation. In children with physical disabilities, the Plaque Index was slightly lower at 2.7 ± 0.6, with a Gingival Index of 2.2 ± 0.5. Children with developmental disorders, such as autism, had the lowest scores, with a Plaque Index of 2.5 ± 0.5 and a Gingival Index of 2.0 ± 0.4, suggesting relatively better, though still suboptimal, and oral hygiene compared to the other groups (Table 3 and Figure 3 see PDF).

Among the 90 children on such medications, the mean DMFT score was 4.6 ± 2.4, with 78% of these children affected by dental caries. In contrast, children not on xerostomia-inducing medications (n=135) had a lower mean DMFT score of 3.1 ± 1.7, and a reduced caries prevalence of 60 % (Table 4).

## Discussion:

The findings of this study reveal a high prevalence of dental caries and poor oral hygiene status among specially-abled children, particularly those with intellectual disabilities. With 67.1% of participants affected by dental caries and a mean DMFT score of 3.8, the results are consistent with previous research, which has also highlighted the increased vulnerability of children with disabilities to oral health issues. This is probably due to reasons such as these: the task of healthily cleaning one's teeth becomes increasingly cumbersome; there are many foods and other substances that are health hazardous, especially with accrued aging; and the effects of some medications that are health-damaging to the teeth. The findings depict a relationship between the number of disabled people and the degree of dental caries, depending on disability type [14]. Findings also showed that children with intellectual disabilities had the highest mean DMFT score of 4.2 and a caries rate of 75% as compared with 62% for children with physical disability and 58% for children with developmental disorders such as autism. This concords with past studies where it have been found that children with intellectual disabilities are likely to be more challenged when it comes to carrying out the basic tasks of brushing their teeth with very much reliance on their carers [15]. As part of the symptoms of ID, children may have certain cognitive and motor deficiencies that may prevent them from performing adequate oral hygiene or from understanding how they should clean their teeth properly thus resulting in a higher prevalence of caries. The study also reveals that all the disability groups have poor oral hygiene status as depicted by higher Plaque Index and Gingival Index values. Participants' mean Plaque Index score which was 2.8 indicated moderate to high levels of plaque formation. However, the highest mean scores have been depicted for plaque (3.0) and gingival health (2.5) among children with an intellectual disability, suggesting that this group is more prone to periodontal disease as well [16]. This has further underlined the role of caregiver support and more specifically oral health promotion by making appropriate interventions among children with disability [17]. The caregivers of these children are often hard-working, dedicated parents who are short on time, knowledge or resources to help with dental care. It is possible to enhance oral hygiene results by introducing individualized educational interventions and reliable recommendations for carers. The results revealed that xerostomia-causing drugs were significantly correlated with higher caries rates [18]. Overall, 78% of children on such medications had caries and a mean DMFT of 4.6, while children not on xerostomia-causing medication had 60% caries experience and mean DMFT of 3.1. This also explains other side effects observed due to a decrease in the flow of saliva, which is responsible for the mouth self-cleaning mechanism and build-up of a calculus or plaque that leads to cavities. The side effects described above should be considered by the healthcare providers when the children with the disabilities are prescribed drugs and prevent the side effects through recommending the use of high fluoride products or saliva substitutes [19, 20]. The study also shows that only a one-third of children brush their teeth twice daily while 15% of children do not even brush their teeth regularly and this information explains the poor oral health status. Surprisingly, the caregivers of the children with intellectual disabilities had comparatively self-reported more challenges in dental care particularly in the care of their child's teeth. It may be helpful for caregiver training initiatives to address the distinct dental needs carrying out by children with special needs and providing modified instruments for oral hygiene care, for instance, toothbrushes using special handles. There are some limitations of this study. Cross-sectional research collects information at one point in time, so we can't analyze changes in oral health status or evaluate the goals of procedures over time. Based on the findings of the current research, there is, therefore, the need to develop and implement more comprehensively sensitive dental care services for children with such disorders. Dental care providers should be trained to address the unique needs of these children, using specialized techniques and tools to enhance the dental care experience. Additionally, preventive strategies such as fluoride treatments, dental sealants, and regular dental check-ups should be prioritized for children with special needs. Among a cohort of 1060 children with physical disabilities, 56.4% (598) were found to have dental caries. The mean DMFT index was calculated to be 1.10, with a standard deviation of ±1.26. The prevalence of dental caries was notably higher in the visually impaired group, recorded at 63.2%, while the hearing and speech impaired group exhibited the lowest prevalence at 51.7%. The recorded oral hygiene status of the study population indicated that 58.5% exhibited good hygiene, 40.8% demonstrated fair hygiene, and 0.7% was classified as having poor hygiene. As a result, it can be said that this group of people needs to be able to easily access dental services and be taught a lot about dental health in order to get the best dental care possible [21]. We found that 65% of children with special healthcare needs had dental caries. The severity classification of dental caries revealed 40% as mild, 20% as moderate, and 5% as severe. Furthermore, 75% of the children demonstrated inadequate oral hygiene, as evidenced by the oral hygiene status assessment. The participant group's mean deft index score of 2.8 indicated a moderate level of dental caries experience. In terms of oral hygiene practices, 60% of participants indicated that they brush their teeth once daily, whereas 40% reported brushing twice daily. Nonetheless, a considerable percentage (70%) indicated the absence of fluoride use, while 55% reported not engaging in regular flossing practices. Consequently, it is concluded that there is a high prevalence of dental caries, inadequate oral hygiene status, and suboptimal oral hygiene habits among children with special healthcare needs in the Jodhpur District [22].

## Conclusion:

Data shows the prevalence of dental caries and poor oral hygiene status is notably high among specially-abled children, particularly those with intellectual disabilities. The findings emphasize the critical need for targeted oral health interventions and caregiver education to address the unique challenges faced by this vulnerable population. Children with intellectual disabilities were found to have the highest DMFT (Decayed, Missing, Filled Teeth) scores and poor oral hygiene, underscoring their increased susceptibility to dental issues. Additionally, the study highlights the significant impact of xerostomia-inducing medications on the prevalence of dental caries.

## Figures and Tables

**Table 1:** Demographic Characteristics of Study Participants (n = 225)

**Demographic Characteristic**	**Number of Children (n)**	**Percentage (%)**
**Age Group (Years)**		
5-8	45	20%
9-12	80	35.60%
13-15	60	26.70%
16-18	40	17.80%
Gender		
Male	130	57.80%
Female	95	42.20%
Type of Disability		
Intellectual Disabilities	102	45.30%
Physical Disabilities	76	33.80%
Developmental Disorders (Autism)	47	20.90%
Medication Use		
On medications causing xerostomia	90	40%
Not on medications causing xerostomia	135	60%

**Table 2:** Prevalence of dental caries by type of disability

**Type of Disability**	**Number of Children (n)**	**Mean DMFT Score (SD)**	**Prevalence of Dental Caries (%)**
Intellectual Disabilities	102	4.2±2.3	75%
Physical Disabilities	76	3.6±1.9	62%
Developmental Disorders (Autism)	47	3.1±1.8	58%

**Table 3:** Oral hygiene status by type of disability

**Type of Disability**	**Plaque Index (Mean ± SD)**	**Gingival Index (Mean ± SD)**
Intellectual Disabilities	3.0± 0.8	2.5± 0.7
Physical Disabilities	2.7± 0.6	2.2± 0.5
Developmental Disorders (Autism)	2.5± 0.5	2.0± 0.4

**Table 4:** Impact of medication use on dental caries prevalence

**Medication Use**	**Number of Children (n)**	**Mean DMFT Score (SD)**	**Prevalence of Dental Caries (%)**
On medications causing xerostomia	90	4.6± 2.4	78%
Not on medications causing xerostomia	135	3.1± 1.7	60%
